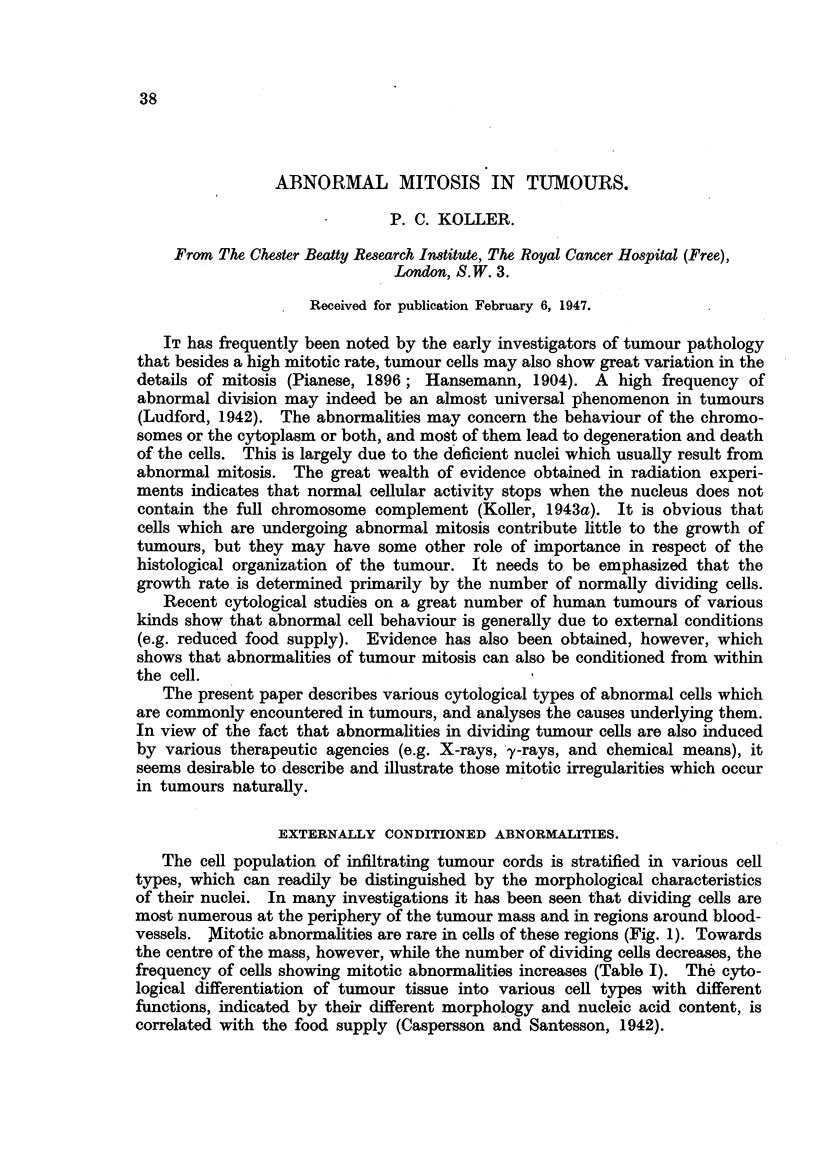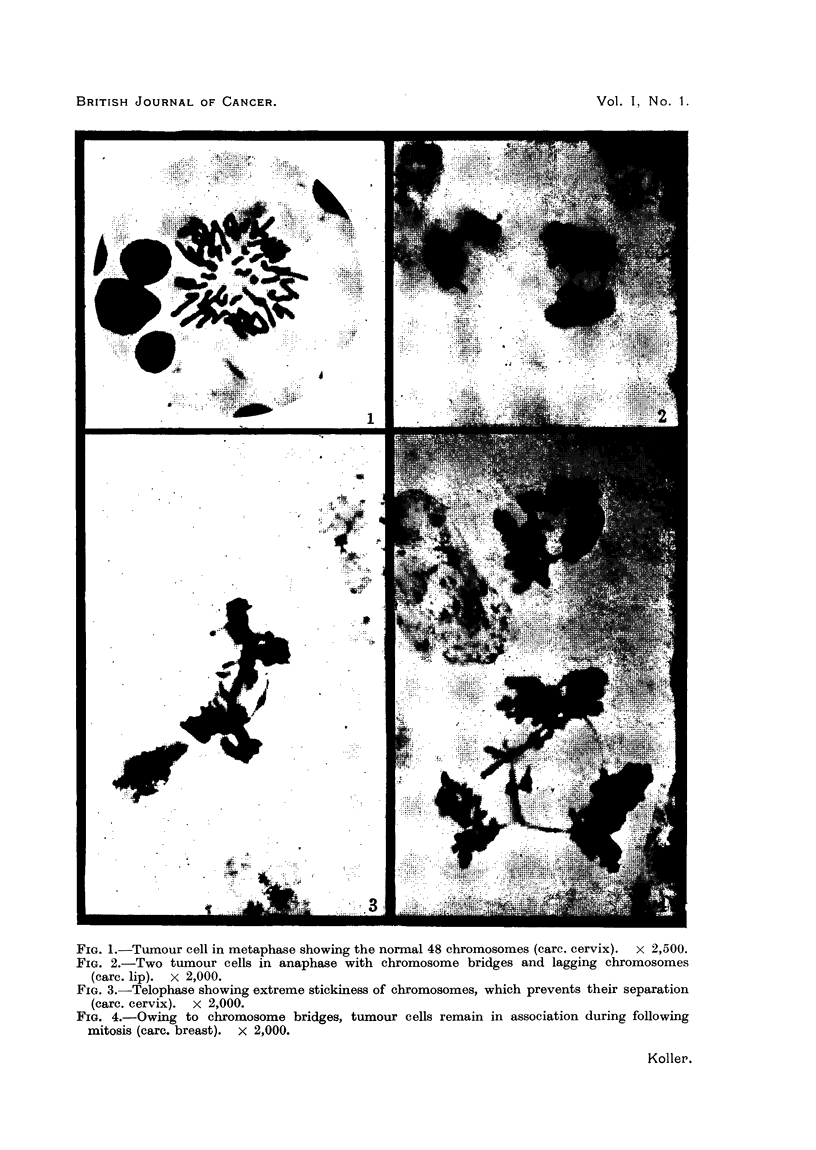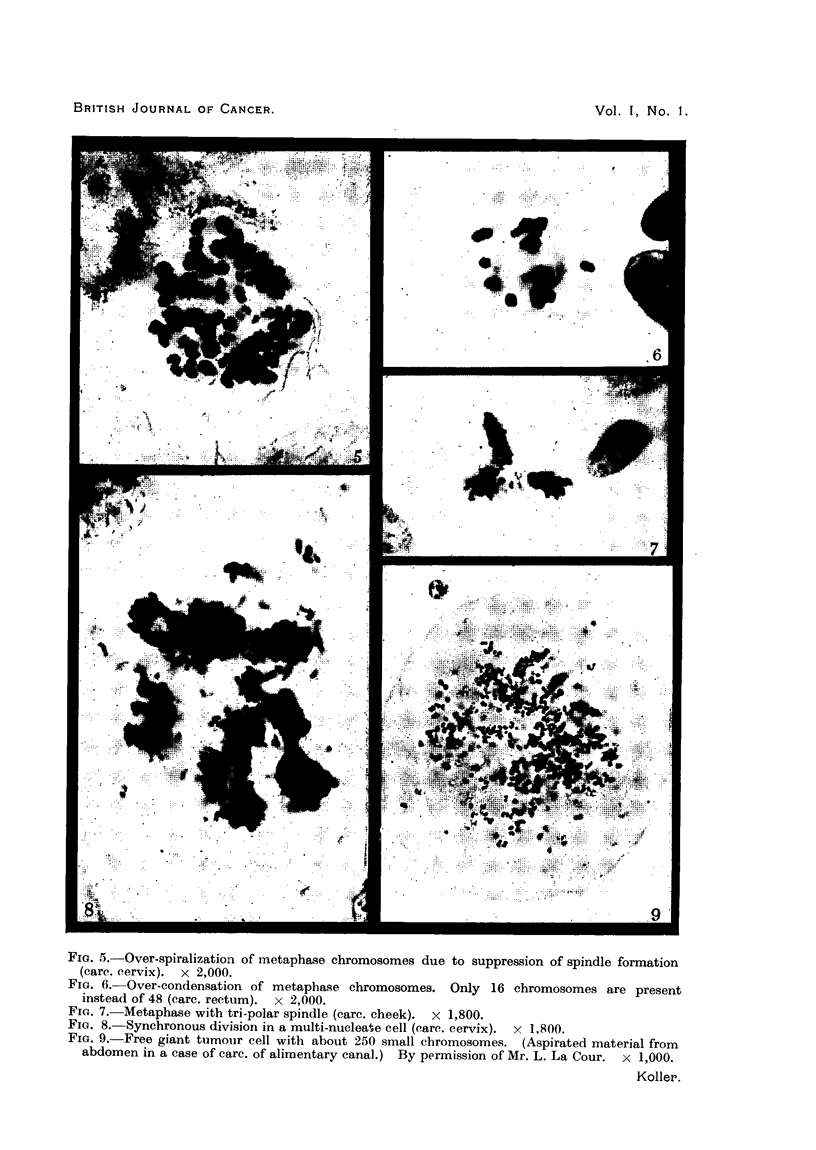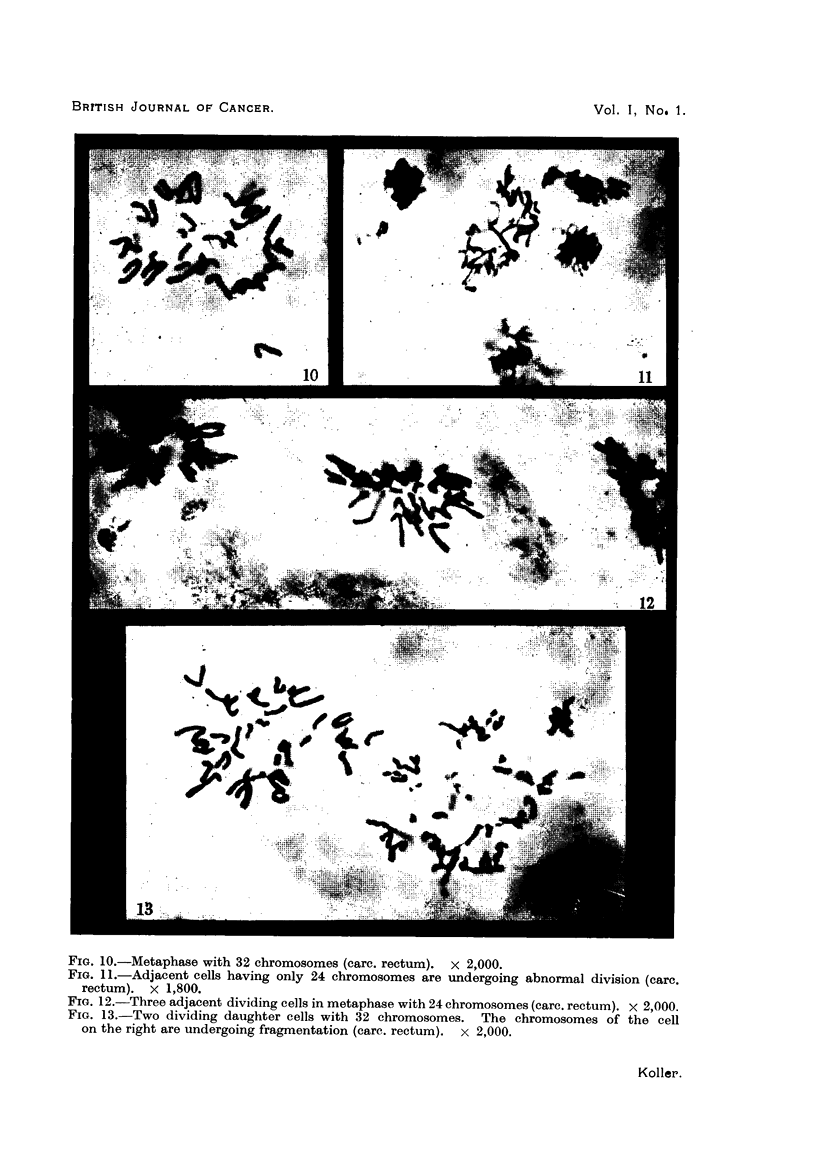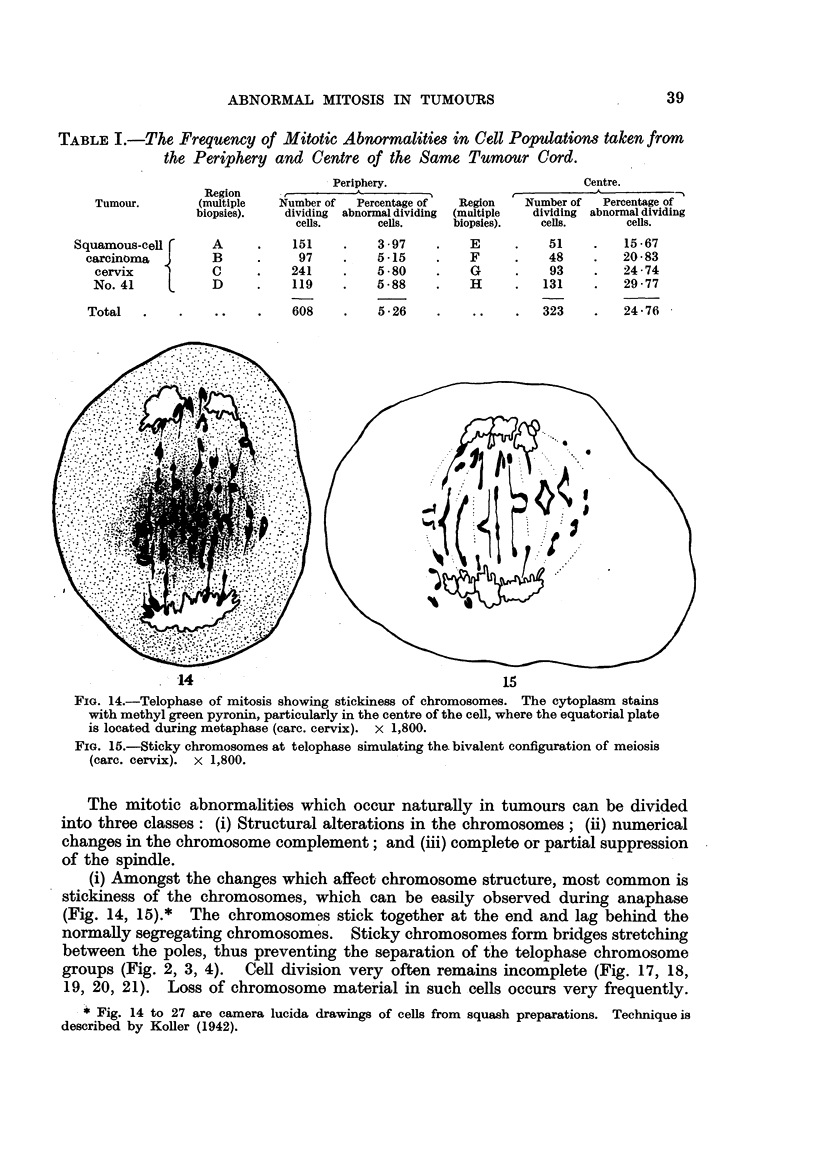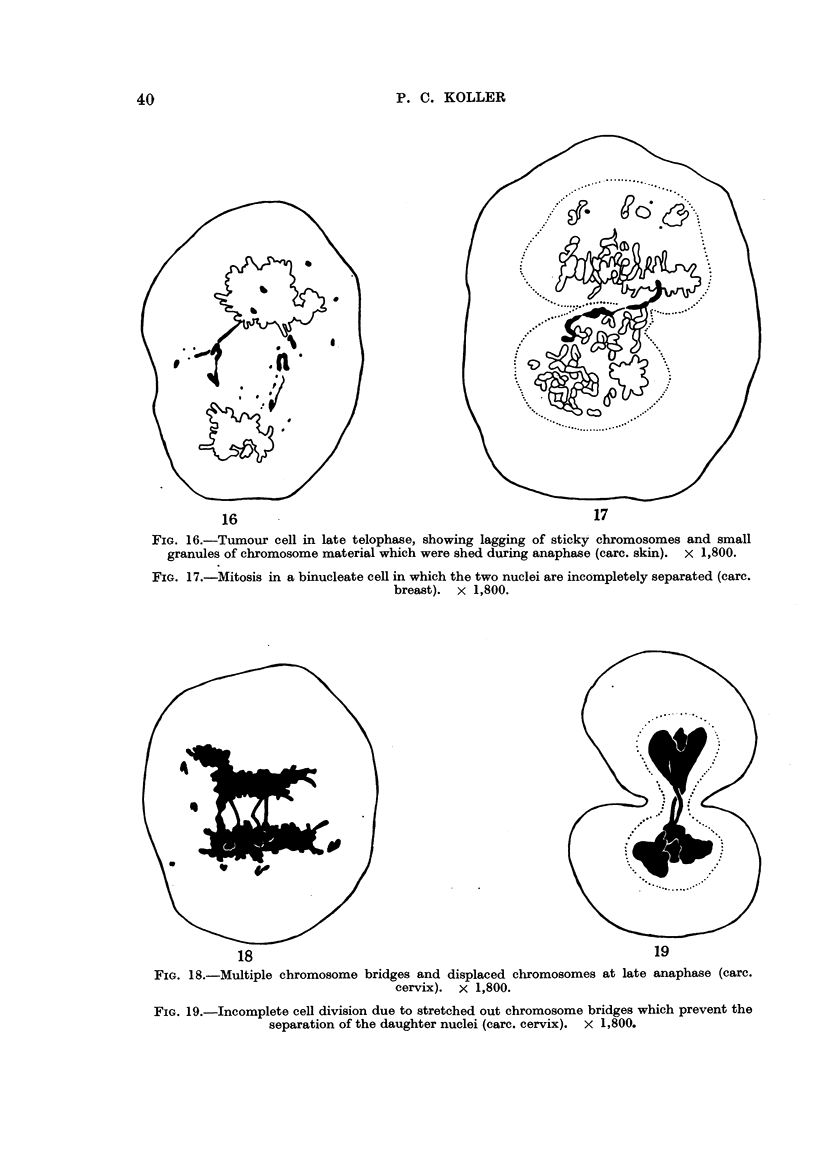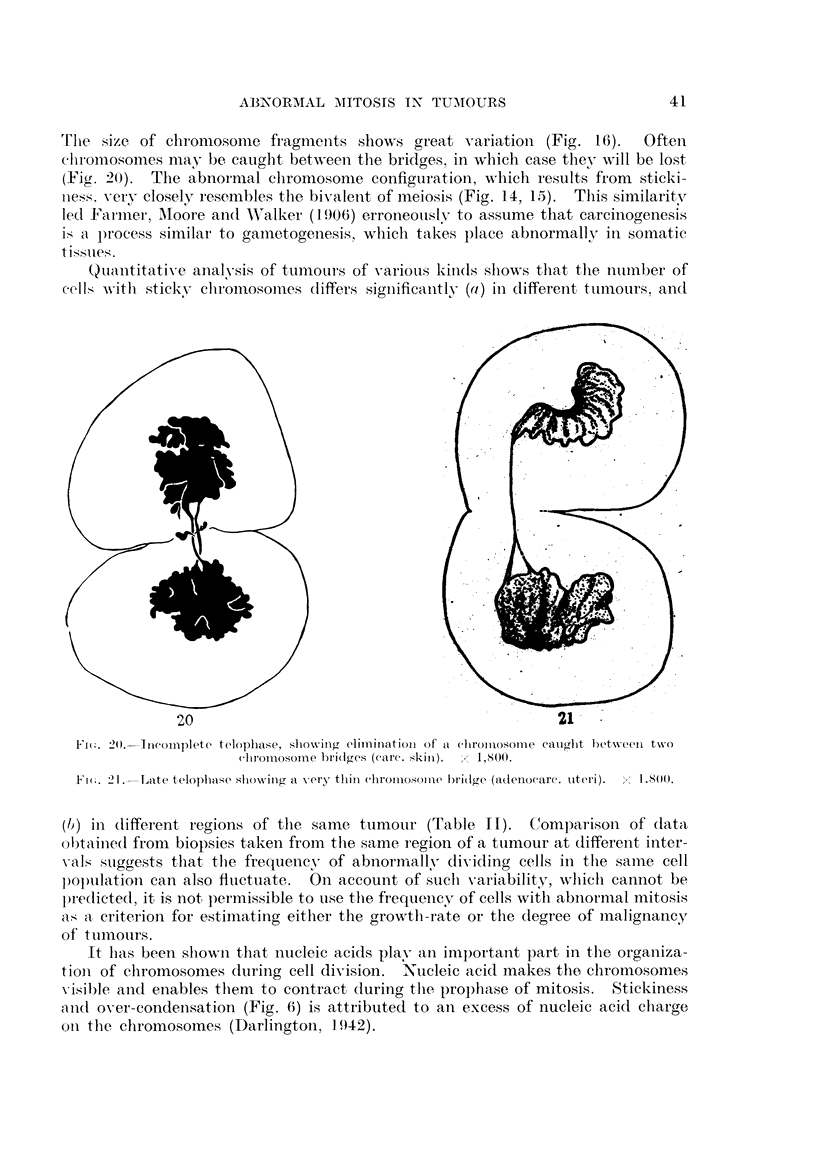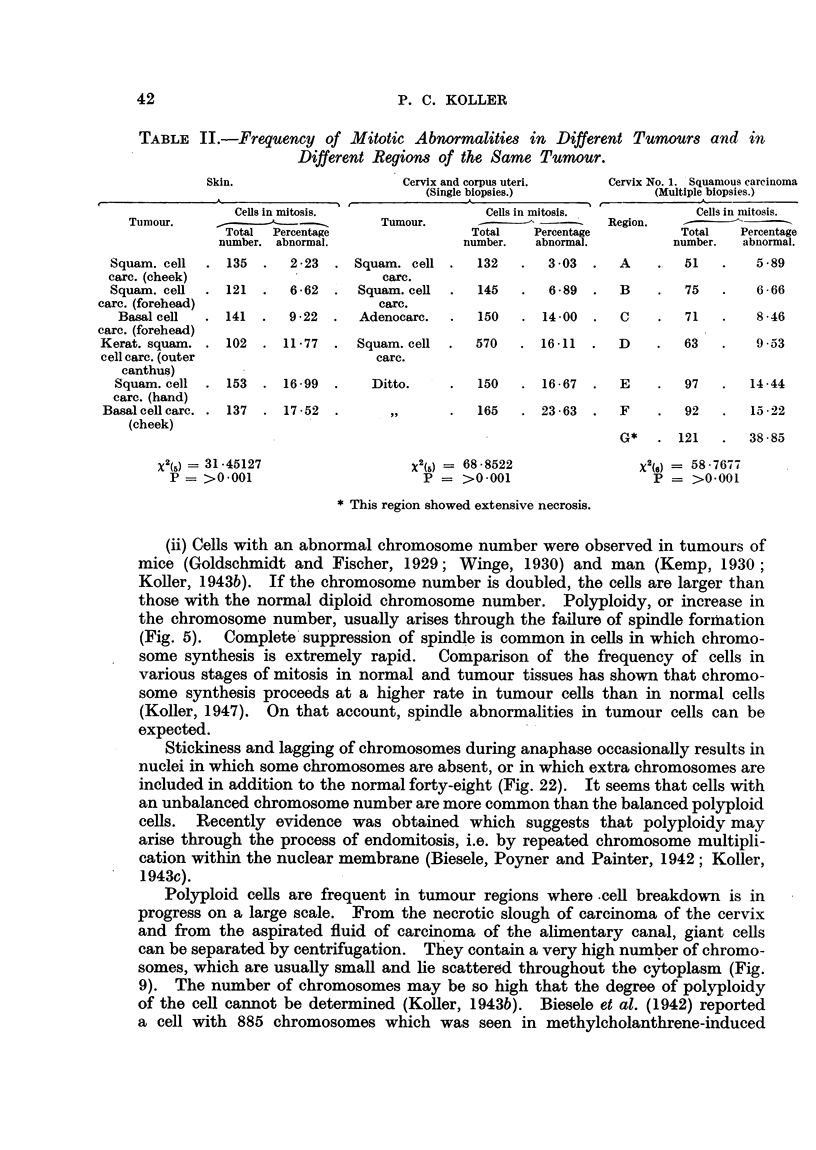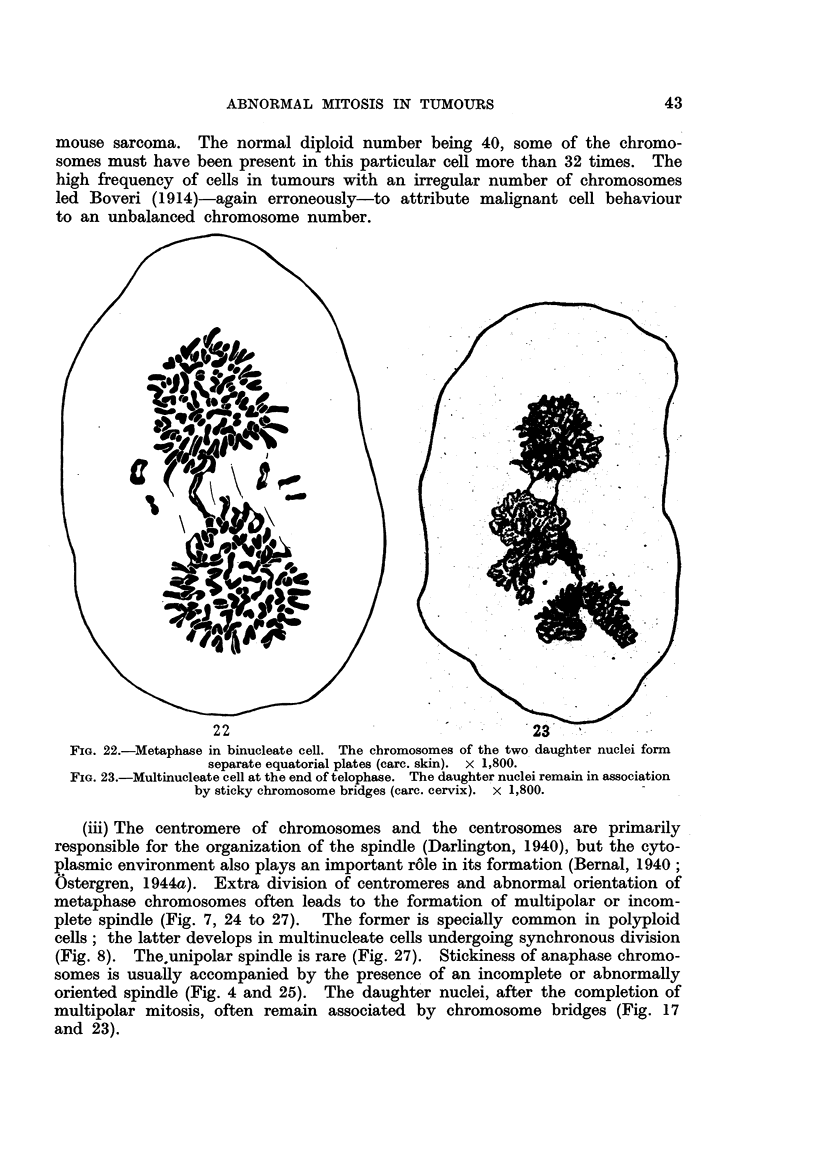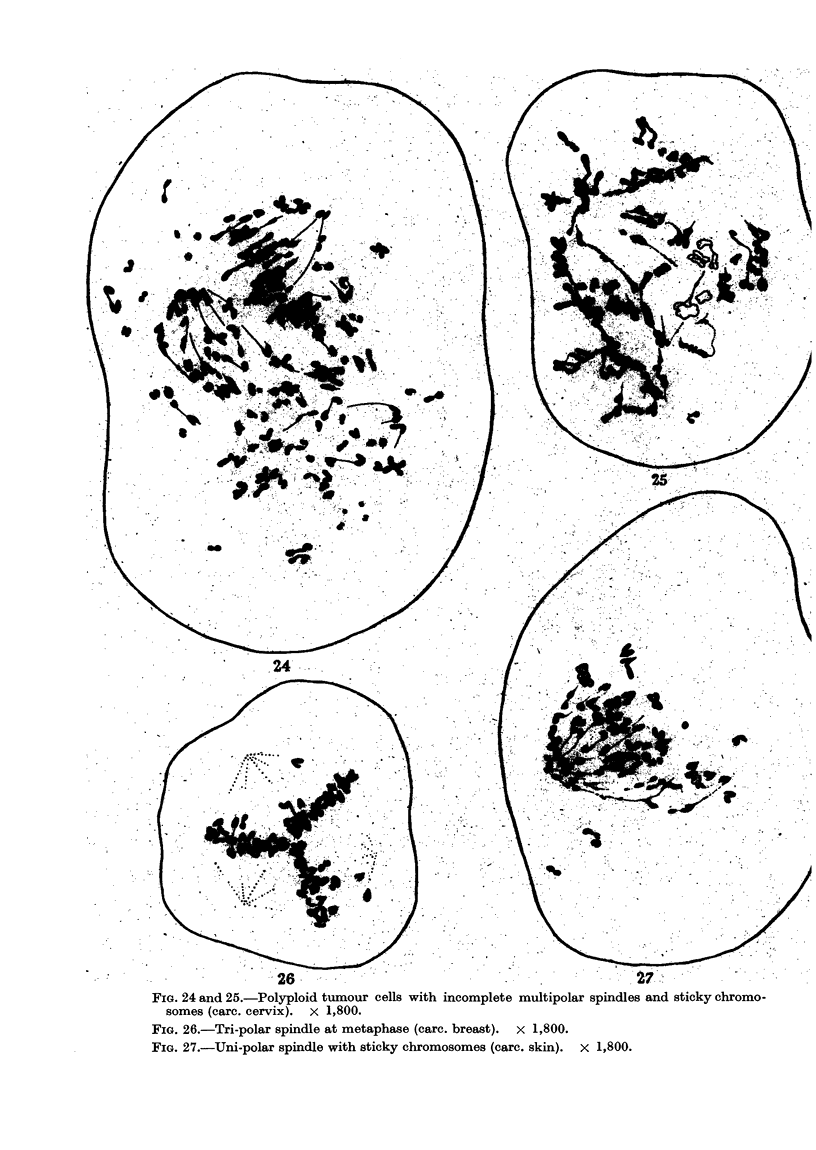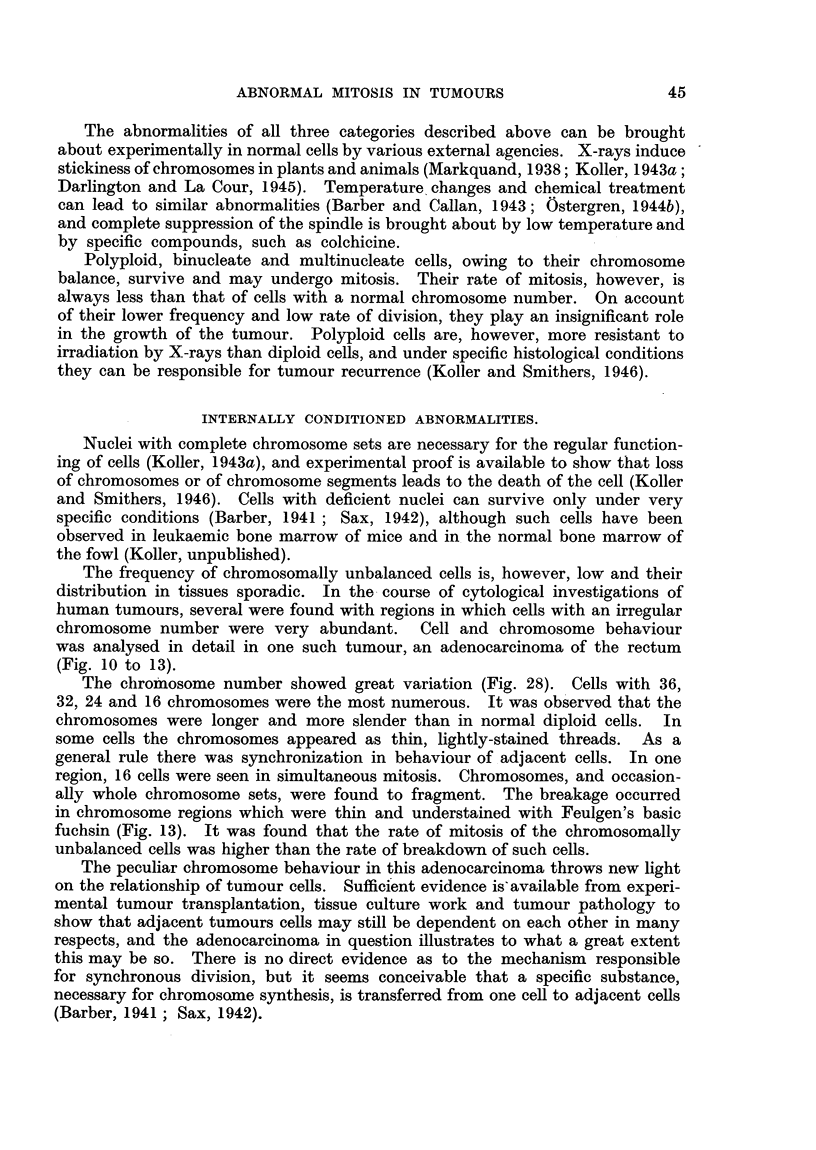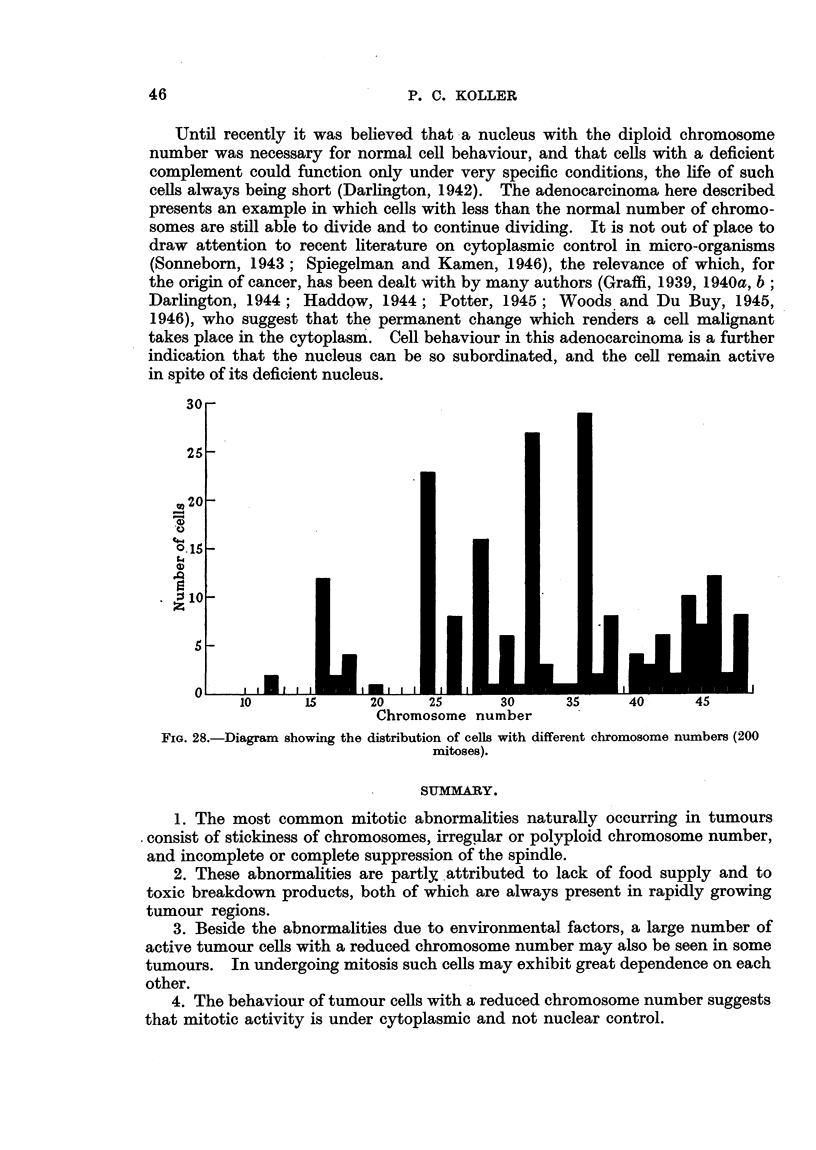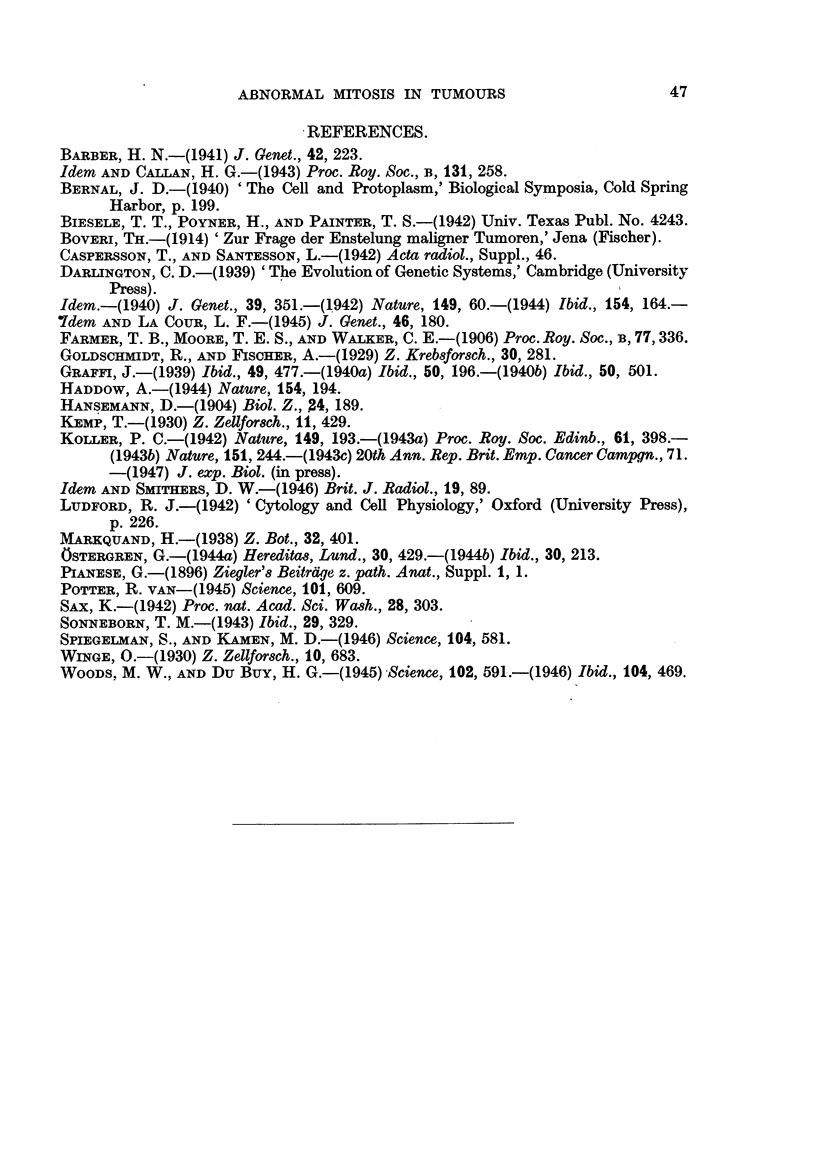# Abnormal Mitosis in Tumours

**DOI:** 10.1038/bjc.1947.5

**Published:** 1947-03

**Authors:** P. C. Koller

## Abstract

**Images:**


### ABNORMAL MITOSIS IN TUMOURS.

P. C. KOLLER.

*From The Chester Beatty Research Institute, The Royal Cancer Hospital (Free), London, S.W. 3.*

Received for publication February 6, 1947.

It has frequently been noted by the early investigators of tumour pathology that besides a high mitotic rate, tumour cells may also show great variation in the details of mitosis (Pianese, 1896; Hansemann, 1904). A high frequency of abnormal division may indeed be an almost universal phenomenon in tumours (Ludford, 1942). The abnormalities may concern the behaviour of the chromosomes or the cytoplasm or both, and most of them lead to degeneration and death of the cells. This is largely due to the deficient nuclei which usually result from abnormal mitosis. The great wealth of evidence obtained in radiation experiments indicates that normal cellular activity stops when the nucleus does not contain the full chromosome complement (Koller, 1943*a*). It is obvious that cells which are undergoing abnormal mitosis contribute little to the growth of tumours, but they may have some other role of importance in respect of the histological organization of the tumour. It needs to be emphasized that the growth rate is determined primarily by the number of normally dividing cells.

Recent cytological studies on a great number of human tumours of various kinds show that abnormal cell behaviour is generally due to external conditions (e.g. reduced food supply). Evidence has also been obtained, however, which shows that abnormalities of tumour mitosis can also be conditioned from within the cell.

The present paper describes various cytological types of abnormal cells which are commonly encountered in tumours, and analyses the causes underlying them. In view of the fact that abnormalities in dividing tumour cells are also induced by various therapeutic agencies (e.g. X-rays, $\gamma$-rays, and chemical means), it seems desirable to describe and illustrate those mitotic irregularities which occur in tumours naturally.

### EXTERNALLY CONDITIONED ABNORMALITIES.

The cell population of infiltrating tumour cords is stratified in various cell types, which can readily be distinguished by the morphological characteristics of their nuclei. In many investigations it has been seen that dividing cells are most numerous at the periphery of the tumour mass and in regions around blood-vessels. Mitotic abnormalities are rare in cells of these regions (Fig. 1). Towards the centre of the mass, however, while the number of dividing cells decreases, the frequency of cells showing mitotic abnormalities increases (Table I). The cytological differentiation of tumour tissue into various cell types with different functions, indicated by their different morphology and nucleic acid content, is correlated with the food supply (Caspersson and Santesson, 1942).

FIG. 1.—Tumour cell in metaphase showing the normal 48 chromosomes (carc. cervix). × 2,500.
FIG. 2.—Two tumour cells in anaphase with chromosome bridges and lagging chromosomes (carc. lip). × 2,000.
FIG. 3.—Telophase showing extreme stickiness of chromosomes, which prevents their separation (carc. cervix). × 2,000.
FIG. 4.—Owing to chromosome bridges, tumour cells remain in association during following mitosis (carc. breast). × 2,000.

Koller.

FIG. 5.—Over-spiralization of metaphase chromosomes due to suppression of spindle formation (carc. cervix). × 2,000.

FIG. 6.—Over-condensation of metaphase chromosomes. Only 16 chromosomes are present instead of 48 (carc. rectum). × 2,000.

FIG. 7.—Metaphase with tri-polar spindle (carc. cheek). × 1,800.

FIG. 8.—Synchronous division in a multi-nucleate cell (carc. cervix). × 1,800.

FIG. 9.—Free giant tumour cell with about 250 small chromosomes. (Aspirated material from abdomen in a case of carc. of alimentary canal.) By permission of Mr. L. La Cour. × 1,000.

Koller.

FIG. 10.—Metaphase with 32 chromosomes (carc. rectum). × 2,000.
FIG. 11.—Adjacent cells having only 24 chromosomes are undergoing abnormal division (carc. rectum). × 1,800.
FIG. 12.—Three adjacent dividing cells in metaphase with 24 chromosomes (carc. rectum). × 2,000.
FIG. 13.—Two dividing daughter cells with 32 chromosomes. The chromosomes of the cell on the right are undergoing fragmentation (carc. rectum). × 2,000.

TABLE I.—*The Frequency of Mitotic Abnormalities in Cell Populations taken from the Periphery and Centre of the Same Tumour Cord.*

| Tumour. | Region (multiple biopsies). | Periphery. Number of dividing cells. | Periphery. Percentage of abnormal dividing cells. | Region (multiple biopsies). | Centre. Number of dividing cells. | Centre. Percentage of abnormal dividing cells. |
|---|---|---|---|---|---|---|
| Squamous-cell carcinoma cervix No. 41 | A | 151 | 3·97 | E | 51 | 15·67 |
| | B | 97 | 5·15 | F | 48 | 20·83 |
| | C | 241 | 5·80 | G | 93 | 24·74 |
| | D | 119 | 5·88 | H | 131 | 29·77 |
| Total | .. | 608 | 5·26 | .. | 323 | 24·76 |

14

15

FIG. 14.—Telophase of mitosis showing stickiness of chromosomes. The cytoplasm stains with methyl green pyronin, particularly in the centre of the cell, where the equatorial plate is located during metaphase (carc. cervix). × 1,800.

FIG. 15.—Sticky chromosomes at telophase simulating the bivalent configuration of meiosis (carc. cervix). × 1,800.

The mitotic abnormalities which occur naturally in tumours can be divided into three classes : (i) Structural alterations in the chromosomes ; (ii) numerical changes in the chromosome complement ; and (iii) complete or partial suppression of the spindle.

(i) Amongst the changes which affect chromosome structure, most common is stickiness of the chromosomes, which can be easily observed during anaphase (Fig. 14, 15).* The chromosomes stick together at the end and lag behind the normally segregating chromosomes. Sticky chromosomes form bridges stretching between the poles, thus preventing the separation of the telophase chromosome groups (Fig. 2, 3, 4). Cell division very often remains incomplete (Fig. 17, 18, 19, 20, 21). Loss of chromosome material in such cells occurs very frequently.

* Fig. 14 to 27 are camera lucida drawings of cells from squash preparations. Technique is described by Koller (1942).

16

17

FIG. 16.—Tumour cell in late telophase, showing lagging of sticky chromosomes and small granules of chromosome material which were shed during anaphase (carc. skin). × 1,800.

FIG. 17.—Mitosis in a binucleate cell in which the two nuclei are incompletely separated (carc. breast). × 1,800.

18

19

FIG. 18.—Multiple chromosome bridges and displaced chromosomes at late anaphase (carc. cervix). × 1,800.

FIG. 19.—Incomplete cell division due to stretched out chromosome bridges which prevent the separation of the daughter nuclei (carc. cervix). × 1,800.

The size of chromosome fragments shows great variation (Fig. 16). Often chromosomes may be caught between the bridges, in which case they will be lost (Fig. 20). The abnormal chromosome configuration, which results from stickiness, very closely resembles the bivalent of meiosis (Fig. 14, 15). This similarity led Farmer, Moore and Walker (1906) erroneously to assume that carcinogenesis is a process similar to gametogenesis, which takes place abnormally in somatic tissues.

Quantitative analysis of tumours of various kinds shows that the number of cells with sticky chromosomes differs significantly (*a*) in different tumours, and

20

21

FIG. 20.—Incomplete telophase, showing elimination of a chromosome caught between two chromosome bridges (carc. skin). × 1,800.

FIG. 21.—Late telophase showing a very thin chromosome bridge (adenocarc. uteri). × 1,800.

(*b*) in different regions of the same tumour (Table II). Comparison of data obtained from biopsies taken from the same region of a tumour at different intervals suggests that the frequency of abnormally dividing cells in the same cell population can also fluctuate. On account of such variability, which cannot be predicted, it is not permissible to use the frequency of cells with abnormal mitosis as a criterion for estimating either the growth-rate or the degree of malignancy of tumours.

It has been shown that nucleic acids play an important part in the organization of chromosomes during cell division. Nucleic acid makes the chromosomes visible and enables them to contract during the prophase of mitosis. Stickiness and over-condensation (Fig. 6) is attributed to an excess of nucleic acid charge on the chromosomes (Darlington, 1942).

TABLE II.—*Frequency of Mitotic Abnormalities in Different Tumours and in Different Regions of the Same Tumour.*

| Skin. | | | Cervix and corpus uteri. (Single biopsies.) | | | Cervix No. 1. Squamous carcinoma (Multiple biopsies.) | | |
|---|---|---|---|---|---|---|---|---|
| Tumour. | Cells in mitosis. Total number. | Cells in mitosis. Percentage abnormal. | Tumour. | Cells in mitosis. Total number. | Cells in mitosis. Percentage abnormal. | Region. | Cells in mitosis. Total number. | Cells in mitosis. Percentage abnormal. |
| Squam. cell carc. (cheek) | 135 | 2·23 | Squam. cell carc. | 132 | 3·03 | A | 51 | 5·89 |
| Squam. cell carc. (forehead) | 121 | 6·62 | Squam. cell carc. | 145 | 6·89 | B | 75 | 6·66 |
| Basal cell carc. (forehead) | 141 | 9·22 | Adenocarc. | 150 | 14·00 | C | 71 | 8·46 |
| Kerat. squam. cell carc. (outer canthus) | 102 | 11·77 | Squam. cell carc. | 570 | 16·11 | D | 63 | 9·53 |
| Squam. cell carc. (hand) | 153 | 16·99 | Ditto. | 150 | 16·67 | E | 97 | 14·44 |
| Basal cell carc. (cheek) | 137 | 17·52 | ,, | 165 | 23·63 | F | 92 | 15·22 |
| | | | | | | G* | 121 | 38·85 |
| $\chi^2_{(5)} = 31 \cdot 45127$, P = >0·001 | | | $\chi^2_{(5)} = 68 \cdot 8522$, P = >0·001 | | | $\chi^2_{(6)} = 58 \cdot 7677$, P = >0·001 | | |

* This region showed extensive necrosis.

(ii) Cells with an abnormal chromosome number were observed in tumours of mice (Goldschmidt and Fischer, 1929; Winge, 1930) and man (Kemp, 1930; Koller, 1943*b*). If the chromosome number is doubled, the cells are larger than those with the normal diploid chromosome number. Polyploidy, or increase in the chromosome number, usually arises through the failure of spindle formation (Fig. 5). Complete suppression of spindle is common in cells in which chromosome synthesis is extremely rapid. Comparison of the frequency of cells in various stages of mitosis in normal and tumour tissues has shown that chromosome synthesis proceeds at a higher rate in tumour cells than in normal cells (Koller, 1947). On that account, spindle abnormalities in tumour cells can be expected.

Stickiness and lagging of chromosomes during anaphase occasionally results in nuclei in which some chromosomes are absent, or in which extra chromosomes are included in addition to the normal forty-eight (Fig. 22). It seems that cells with an unbalanced chromosome number are more common than the balanced polyploid cells. Recently evidence was obtained which suggests that polyploidy may arise through the process of endomitosis, i.e. by repeated chromosome multiplication within the nuclear membrane (Biesele, Poyner and Painter, 1942; Koller, 1943*c*).

Polyploid cells are frequent in tumour regions where cell breakdown is in progress on a large scale. From the necrotic slough of carcinoma of the cervix and from the aspirated fluid of carcinoma of the alimentary canal, giant cells can be separated by centrifugation. They contain a very high number of chromosomes, which are usually small and lie scattered throughout the cytoplasm (Fig. 9). The number of chromosomes may be so high that the degree of polyploidy of the cell cannot be determined (Koller, 1943*b*). Biesele *et al.* (1942) reported a cell with 885 chromosomes which was seen in methylcholanthrene-induced mouse sarcoma. The normal diploid number being 40, some of the chromosomes must have been present in this particular cell more than 32 times. The high frequency of cells in tumours with an irregular number of chromosomes led Boveri (1914)—again erroneously—to attribute malignant cell behaviour to an unbalanced chromosome number.

22

23

FIG. 22.—Metaphase in binucleate cell. The chromosomes of the two daughter nuclei form separate equatorial plates (carc. skin). × 1,800.

FIG. 23.—Multinucleate cell at the end of telophase. The daughter nuclei remain in association by sticky chromosome bridges (carc. cervix). × 1,800.

(iii) The centromere of chromosomes and the centrosomes are primarily responsible for the organization of the spindle (Darlington, 1940), but the cytoplasmic environment also plays an important rôle in its formation (Bernal, 1940 ; Östergren, 1944*a*). Extra division of centromeres and abnormal orientation of metaphase chromosomes often leads to the formation of multipolar or incomplete spindle (Fig. 7, 24 to 27). The former is specially common in polyploid cells ; the latter develops in multinucleate cells undergoing synchronous division (Fig. 8). The unipolar spindle is rare (Fig. 27). Stickiness of anaphase chromosomes is usually accompanied by the presence of an incomplete or abnormally oriented spindle (Fig. 4 and 25). The daughter nuclei, after the completion of multipolar mitosis, often remain associated by chromosome bridges (Fig. 17 and 23).

24

25

26

27

Fig. 24 and 25.—Polyploid tumour cells with incomplete multipolar spindles and sticky chromosomes (carc. cervix). × 1,800.

Fig. 26.—Tri-polar spindle at metaphase (carc. breast). × 1,800.

Fig. 27.—Uni-polar spindle with sticky chromosomes (carc. skin). × 1,800.

The abnormalities of all three categories described above can be brought about experimentally in normal cells by various external agencies. X-rays induce stickiness of chromosomes in plants and animals (Markquand, 1938; Koller, 1943*a*; Darlington and La Cour, 1945). Temperature changes and chemical treatment can lead to similar abnormalities (Barber and Callan, 1943; Östergren, 1944*b*), and complete suppression of the spindle is brought about by low temperature and by specific compounds, such as colchicine.

Polyploid, binucleate and multinucleate cells, owing to their chromosome balance, survive and may undergo mitosis. Their rate of mitosis, however, is always less than that of cells with a normal chromosome number. On account of their lower frequency and low rate of division, they play an insignificant role in the growth of the tumour. Polyploid cells are, however, more resistant to irradiation by X-rays than diploid cells, and under specific histological conditions they can be responsible for tumour recurrence (Koller and Smithers, 1946).

### INTERNALLY CONDITIONED ABNORMALITIES.

Nuclei with complete chromosome sets are necessary for the regular functioning of cells (Koller, 1943*a*), and experimental proof is available to show that loss of chromosomes or of chromosome segments leads to the death of the cell (Koller and Smithers, 1946). Cells with deficient nuclei can survive only under very specific conditions (Barber, 1941; Sax, 1942), although such cells have been observed in leukaemic bone marrow of mice and in the normal bone marrow of the fowl (Koller, unpublished).

The frequency of chromosomally unbalanced cells is, however, low and their distribution in tissues sporadic. In the course of cytological investigations of human tumours, several were found with regions in which cells with an irregular chromosome number were very abundant. Cell and chromosome behaviour was analysed in detail in one such tumour, an adenocarcinoma of the rectum (Fig. 10 to 13).

The chromosome number showed great variation (Fig. 28). Cells with 36, 32, 24 and 16 chromosomes were the most numerous. It was observed that the chromosomes were longer and more slender than in normal diploid cells. In some cells the chromosomes appeared as thin, lightly-stained threads. As a general rule there was synchronization in behaviour of adjacent cells. In one region, 16 cells were seen in simultaneous mitosis. Chromosomes, and occasionally whole chromosome sets, were found to fragment. The breakage occurred in chromosome regions which were thin and understained with Feulgen's basic fuchsin (Fig. 13). It was found that the rate of mitosis of the chromosomally unbalanced cells was higher than the rate of breakdown of such cells.

The peculiar chromosome behaviour in this adenocarcinoma throws new light on the relationship of tumour cells. Sufficient evidence is available from experimental tumour transplantation, tissue culture work and tumour pathology to show that adjacent tumours cells may still be dependent on each other in many respects, and the adenocarcinoma in question illustrates to what a great extent this may be so. There is no direct evidence as to the mechanism responsible for synchronous division, but it seems conceivable that a specific substance, necessary for chromosome synthesis, is transferred from one cell to adjacent cells (Barber, 1941; Sax, 1942).

Until recently it was believed that a nucleus with the diploid chromosome number was necessary for normal cell behaviour, and that cells with a deficient complement could function only under very specific conditions, the life of such cells always being short (Darlington, 1942). The adenocarcinoma here described presents an example in which cells with less than the normal number of chromosomes are still able to divide and to continue dividing. It is not out of place to draw attention to recent literature on cytoplasmic control in micro-organisms (Sonneborn, 1943; Spiegelman and Kamen, 1946), the relevance of which, for the origin of cancer, has been dealt with by many authors (Graffi, 1939, 1940*a*, *b*; Darlington, 1944; Haddow, 1944; Potter, 1945; Woods and Du Buy, 1945, 1946), who suggest that the permanent change which renders a cell malignant takes place in the cytoplasm. Cell behaviour in this adenocarcinoma is a further indication that the nucleus can be so subordinated, and the cell remain active in spite of its deficient nucleus.

FIG. 28.—Diagram showing the distribution of cells with different chromosome numbers (200 mitoses).

SUMMARY.

1. The most common mitotic abnormalities naturally occurring in tumours consist of stickiness of chromosomes, irregular or polyploid chromosome number, and incomplete or complete suppression of the spindle.

2. These abnormalities are partly attributed to lack of food supply and to toxic breakdown products, both of which are always present in rapidly growing tumour regions.

3. Beside the abnormalities due to environmental factors, a large number of active tumour cells with a reduced chromosome number may also be seen in some tumours. In undergoing mitosis such cells may exhibit great dependence on each other.

4. The behaviour of tumour cells with a reduced chromosome number suggests that mitotic activity is under cytoplasmic and not nuclear control.

### REFERENCES.

BARBER, H. N.—(1941) *J. Genet.*, **42**, 223.
*Idem* AND CALLAN, H. G.—(1943) *Proc. Roy. Soc.*, B, **131**, 258.
BERNAL, J. D.—(1940) 'The Cell and Protoplasm,' Biological Symposia, Cold Spring Harbor, p. 199.
BIESELE, T. T., POYNER, H., AND PAINTER, T. S.—(1942) Univ. Texas Publ. No. 4243.
BOVERI, TH.—(1914) 'Zur Frage der Enstelung maligner Tumoren,' Jena (Fischer).
CASPERSSON, T., AND SANTESSON, L.—(1942) *Acta radiol.*, Suppl., 46.
DARLINGTON, C. D.—(1939) 'The Evolution of Genetic Systems,' Cambridge (University Press).
*Idem.*—(1940) *J. Genet.*, **39**, 351.—(1942) *Nature*, **149**, 60.—(1944) *Ibid.*, **154**, 164.—
*Idem* AND LA COUR, L. F.—(1945) *J. Genet.*, **46**, 180.
FARMER, T. B., MOORE, T. E. S., AND WALKER, C. E.—(1906) *Proc. Roy. Soc.*, B, **77**, 336.
GOLDSCHMIDT, R., AND FISCHER, A.—(1929) *Z. Krebsforsch.*, **30**, 281.
GRAFFI, J.—(1939) *Ibid.*, **49**, 477.—(1940*a*) *Ibid.*, **50**, 196.—(1940*b*) *Ibid.*, **50**, 501.
HADDOW, A.—(1944) *Nature*, **154**, 194.
HANSEMANN, D.—(1904) *Biol. Z.*, **24**, 189.
KEMP, T.—(1930) *Z. Zellforsch.*, **11**, 429.
KOLLER, P. C.—(1942) *Nature*, **149**, 193.—(1943*a*) *Proc. Roy. Soc. Edinb.*, **61**, 398.—(1943*b*) *Nature*, **151**, 244.—(1943*c*) *20th Ann. Rep. Brit. Emp. Cancer Campgn.*, 71.—(1947) *J. exp. Biol.* (in press).
*Idem* AND SMITHERS, D. W.—(1946) *Brit. J. Radiol.*, **19**, 89.
LUDFORD, R. J.—(1942) 'Cytology and Cell Physiology,' Oxford (University Press), p. 226.
MARKQUAND, H.—(1938) *Z. Bot.*, **32**, 401.
ÖSTERGREN, G.—(1944*a*) *Hereditas, Lund.*, **30**, 429.—(1944*b*) *Ibid.*, **30**, 213.
PIANESE, G.—(1896) *Ziegler's Beiträge z. path. Anat.*, Suppl. **1**, 1.
POTTER, R. VAN—(1945) *Science*, **101**, 609.
SAX, K.—(1942) *Proc. nat. Acad. Sci. Wash.*, **28**, 303.
SONNEBORN, T. M.—(1943) *Ibid.*, **29**, 329.
SPIEGELMAN, S., AND KAMEN, M. D.—(1946) *Science*, **104**, 581.
WINGE, O.—(1930) *Z. Zellforsch.*, **10**, 683.
WOODS, M. W., AND DU BUY, H. G.—(1945) *Science*, **102**, 591.—(1946) *Ibid.*, **104**, 469.